# Prevalence, Pattern and Predictors for Dyslipidemia of Chinese Women With Polycystic Ovary Syndrome

**DOI:** 10.3389/fcvm.2021.790454

**Published:** 2021-12-15

**Authors:** Xi Luo, Wang-Yu Cai, Xiao-Ke Wu

**Affiliations:** ^1^Department of Obstetrics and Gynecology, The Second Affiliated Hospital of Zhejiang Chinese Medical University, Hangzhou, China; ^2^Fourth Affiliated Hospital, Zhejiang University School of Medicine, Yiwu, China; ^3^Department of Gynecology, First Affiliated Hospital, Heilongjiang University of Chinese Medicine, Harbin, China; ^4^Heilongjiang Province Hospital, Harbin Institute of Technology, Harbin, China

**Keywords:** polycystic ovary syndrome, dyslipidemia, prevalence, predictor, HDL

## Abstract

**Objective:** To investigate the prevalence, pattern and risk predictors for dyslipidemia among Chinese women with polycystic ovary syndrome (PCOS).

**Study Design and Methods:** A total of 1,000 women diagnosed as PCOS by modified Rotterdam criteria were enrolled in 27 hospitals across China in a randomized controlled trial. Anthropometric, metabolic parameters, sex hormone, and lipid levels were measured at the baseline visit. Dyslipidemia was defined according to total cholesterol (TC), low density lipoprotein cholesterol (LDL-C), high-density lipoprotein cholesterol (HDL-C), and triglycerides (TG) level. Independent *t*-test and logistic regression were used to identify predictors for dyslipidemia. Area under the receiver operating characteristic curve (AUC) was calculated.

**Results:** A total of 41.3% of the women had dyslipidemia, and the prevalence of abnormal TC, LDL-C, HDL-C, and TG were 8.6, 9.1, 26.9, and 17.5%, respectively. Logistic regression found that age, waist circumference, insulin, follicle-stimulating hormone, and sex hormone-binding globulin were independent predictors for dyslipidemia. When combining these predictors, the AUC was 0.744. The cut-off points were age >28.5 years, waist circumference >86.5 cm, insulin >96.0 pmol/L, follicle-stimulating hormone <5.6 mIU/mL, and sex hormone-binding hormone <31.0 nmol/L, respectively.

**Conclusion:** Dyslipidemia was common in Chinese women with PCOS, and low HDL-C level was the predominant lipid abnormality. Age, waist circumference, follicle-stimulating hormone, insulin and sex hormone-binding globulin were predictive for dyslipidemia among Chinese women with PCOS.

## Introduction

Cardiovascular disease (CVD) is the leading cause of death globally (1). Dyslipidemia is a major risk factor for development and progression of CVD. Previous studies have reported that high levels of total cholesterol (TC), low-density lipoprotein cholesterol (LDL-C), and triglycerides (TG) and low level of high-density lipoprotein cholesterol (HDL-C) are associated with increased risk of CVD (2–5).

Polycystic ovary syndrome (PCOS) is one of the most common reproductive disorders in childbearing-aged women, manifested with menstrual disorder, hyperandrogenism, infertility, and polycystic ovary morphology (6). PCOS presented a cluster of metabolic abnormalities that are linked with an increased risk of CVD (7). Dyslipidemia is also common in PCOS. In a meta-analysis, Wild et al. reported that TG levels were 26 mg/dL higher, LDL-C were 12 mg/dL higher, non-HDL-C concentrations were 19 mg/dL higher, and HDL-C concentrations were 6 mg/dL lower in women with PCOS than those of controls (8). The prevalence of dyslipidemia is also higher in women with PCOS compared to control women (9), and up to 50–70% in some studies (10, 11). Furthermore, more research found that dyslipidemia is related to female reproductive health. Serum lipid levels were associated with clinical pregnancy, live birth and miscarriage in women undergoing assisted reproduction (12). Previous study showed that abnormal TC, TG, LDL-C, and HDL-C levels were associated with increased number of oocytes retrieved in women with PCOS undergoing unstimulated natural cycles (13). High serum TC was also a risk factor for reproductive outcomes of PCOS patients undergoing assisted reproduction (14). Therefore, screening for dyslipidemia may not only help to evaluate the cardiometabolic health, but also useful to predict reproductive outcomes after fertility treatment for women with PCOS.

To date, there were few studies focused on the dyslipidemia of Chinese women with PCOS. Therefore, we aimed to determine the prevalence, pattern, and predictors for dyslipidemia in a cohort of Chinese women with PCOS from a well-designed randomized controlled trial.

## Methods

### Design

This is a baseline cross-sectional analysis of a randomized controlled trial in China, which was published elsewhere (15). Patients were recruited from July 2012 to November 2014. Total 1,000 women were included in 27 tertiary or secondary hospitals across China mainland. This trial was registered on ClinicalTrials.gov (NCT01573858) and chictr.org.cn (ChiCTR-TRC-12002081). The trial protocol was approved in ethical committees in all local sites. All patients signed an informed consent. Details of the trial can be found in the main article of this trial (15).

### Patients

In this trial, all participants were diagnosed as PCOS by the modified Rotterdam criteria (6), which is also Chinese version of PCOS diagnosis criteria (16). Participants were required to have oligomenorrhea (defined as a menstrual interval >35 days or <8 menses in the past year) or amenorrhea (define as a menstrual interval >90 days), together with hyperandrogenism [clinical hyperandrogenism: modified Ferriman-Gallwey hirsutism score ≥ 5 (17), biochemical hyperandrogenism: serum total testosterone >1.67 nmol/L], polycystic ovaries morphology in ultrasound (≥ 12 antral follicles 2–9 mm or ovarian volume ≥ 10 cm^3^) or both. Patients with metabolic diseases including type one or type two diabetes, any liver and renal diseases were excluded.

### Measurements

At the baseline visit, all participants underwent a comprehensive physical evaluation in a standard method by a research assistant. Height, weight, waist circumference (WC), hip circumference (HC), systolic blood pressure (SBP) and diastolic blood pressure (DBP) were measured and body mass index (BMI) was calculated by height and weight by the following formulas: BMI = weight(kg)/height(m)^2^.

Blood samples were collected at day 3 in the menstrual cycle for each participant at baseline visit. All blood samples were shipped back to the core laboratory at Heilongjiang University of Chinese Medicine for measurement. Metabolic and endocrinologic parameter including glucose, insulin, progesterone, testosterone, luteinizing hormone (LH), follicle-stimulating hormone (FSH), estradiol (E2), total testosterone, and sex hormone-binding globulin (SHBG) were measured. Homeostatic model assessment-insulin resistance (HOMA-IR) was calculated by glucose and insulin by the following formulas: HOMA-IR = [*insulin*(*mIU*/*L*) × *glucose*(*mmol*/*L*)]/22.5. Lipids panel included HDL-C, LDL-C, TG, TC, apolipoprotein A1 (APOA1), apolipoprotein B (APOB), and lipoprotein (LP).

### Definition of Dyslipidemia

Dyslipidemia was defined in accordance with the Chinese Guidelines on Prevention and Treatment of Dyslipidemia in Adults as follows: TC levels ≥ 6.22 mmol/L, TG levels ≥ 2.26 mmol/L, LDL-C levels ≥ 4.14 mmol/L, and HDL-C levels ≤ 1.04 mmol/L (18).

### Laboratory Assessment

HDL-C and LDL-C were measured by direct-method assays, and TG and TC were measured by the N-(3-sulfopropyl)-3-methoxy-5-methylaniline method (Wako Diagnostics). Serum APOA1 and APOB levels were determined by the polyethylene glycol-enhanced immunoturbidimetric assay (Maker). Glucose was measured with a hexokinase assay (Maker), insulin, total testosterone, E2, progesterone, FSH, and LH were analyzed with an electrochemiluminescence immunoassay (Roche Diagnostics). SHBG was measured by chemiluminescence immunoassay (Siemens).

### Statistical Analysis

All data were analyzed using SPSS Statistics 25.0 version (IBM SPSS, Inc., Chicago, IL, USA). Data were presented as mean ± standard deviation or number with frequency. Independent *t*-test and chi-square test were used to compare between groups. Logistic regression analysis was performed to explore the association between potential predictors for dyslipidemia. The receiver operating characteristic (ROC) curve and area under the curve (AUC) was used to evaluate the predictive ability for dyslipidemia. The cut-off point of a predictor was defined as the value with highest sum of sensitivity and specificity. *P* < 0.05 was considered statistically significant.

## Results

**Figure 1:**
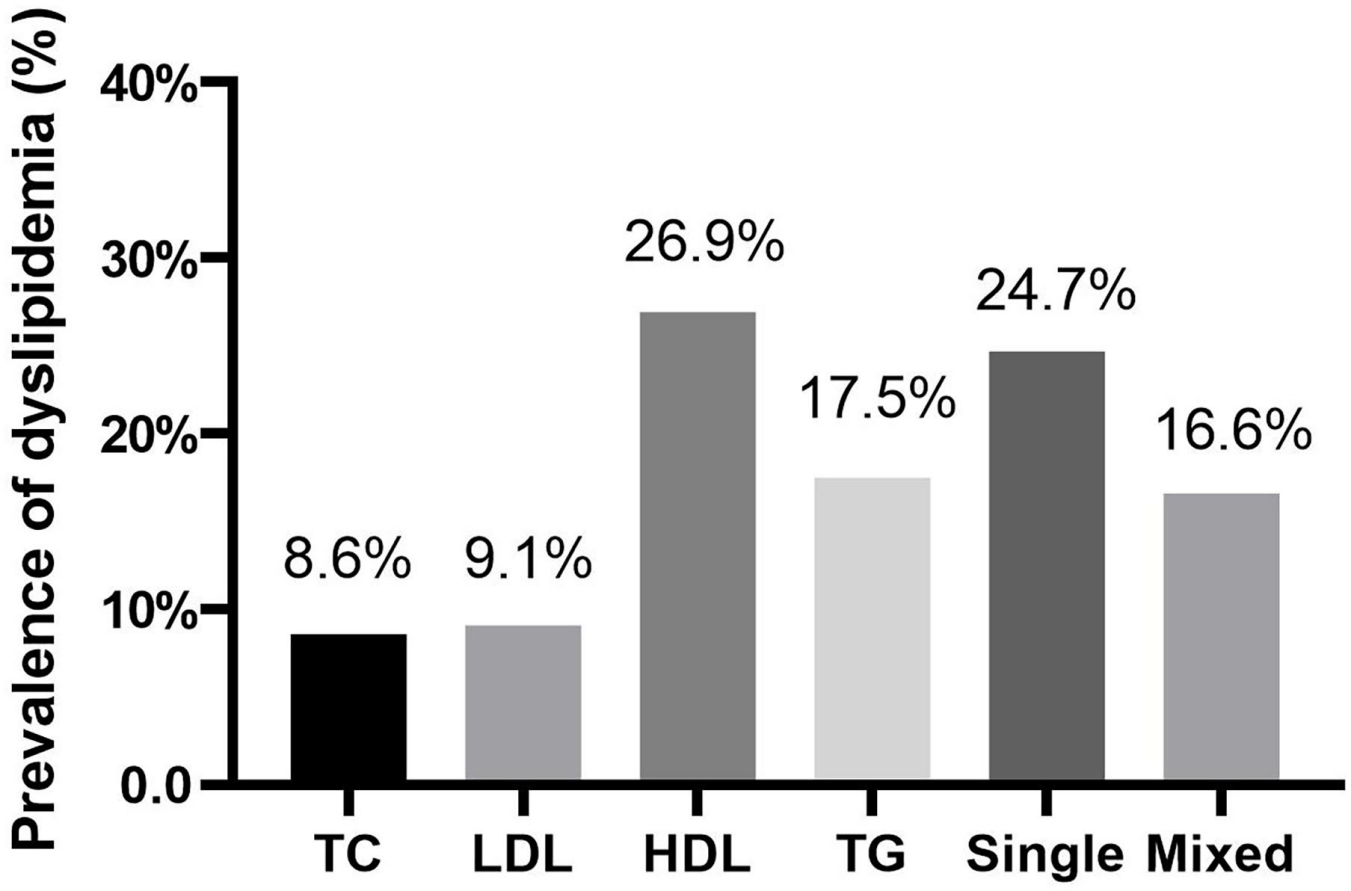
The prevlance of dyslipidemia among Chinese women with polycystic ovary syndrome. TC, ≥ 6.22 mmol/L; LDL-C, ≥ 4.14 mmol/L; HDL-C, ≤ 1.04 mmol/L; TG, ≥ 2.26 mmol/L; Single, presented only one type of dyslipidemia; Mix, presented more than one type of dyslipidemia.

A total of 1,000 Chinese women with PCOS were included. The women had a mean age of 27.9 ± 3.3 years, an average BMI of 24.2 ± 4.3 kg/m^2^. The mean values of TC, LDL-C, HDL-C, and TG were 4.7 ± 1.1, 3.0 ± 0.9, 1.3 ± 0.4, and 1.6 ± 0.9 mmol/L, respectively. A total of 413 (41.3%) of these women were diagnosed with dyslipidemia. The prevalence of abnormal TC, LDL-C, HDL-C, and TG were 8.6, 9.1, 26.9, and 17.5%, respectively. 24.7% of women had only one type of lipid abnormality and 16.6% of women had more than one type of lipid abnormality (Figure 1).

Table 1 demonstrated the baseline characteristics of all 1,000 women and women with and without dyslipidemia. PCOS women with dyslipidemia had increased age, BMI, WC, HC, SBP, DBP, duration of infertility, insulin, HOMA-IR, lower LH, FSH, LH/FSH ratio and SHBG compared to women without dyslipidemia.

**Table 1:** Characteristics of all women and women with and without dyslipidemia.

**Characteristics**	**Total PCOS women** **(***n*** = 1,000)**	**With dyslipidemia** **(***n*** = 413)**	**Without dyslipidemia** **(***n*** = 587)**	***P*** value*
Age (year)	27.9 ± 3.3	28.4 ± 3.5	27.5 ± 3.1	<0.001
BMI (kg/m^2^)	24.2 ± 4.3	25.7 ± 4.1	23.1 ± 4.1	<0.001
HC (cm)	98.5 ± 8.6	100.8 ± 8.6	96.5 ± 8.1	<0.001
WC (cm)	85.4 ± 11.5	89.4 ± 11.1	82.4 ± 10.8	<0.001
SBP (mmHg)	112.3 ± 9.4	113.6 ± 9.0	111.3 ± 9.6	<0.001
DBP (mmHg)	74.9 ± 7.9	75.4 ± 7.9	74.4 ± 7.8	0.049
Duration of infertility (month)	24.0 ± 17.8	25.8 ± 18.8	22.4 ± 16.5	0.004
Current smoking	12 (1.2%)	8 (1.9%)	4 (0.7%)	0.098
Current drinking	91 (9.1%)	42 (10.2%)	48 (8.2%)	0.423
Insulin (pmol/L)	98.1 ± 108.5	124.8 ± 135.4	76.4 ± 70.9	<0.001
Glucose (mmol/L)	5.0 ± 1.0	5.1 ± 1.2	5.0 ± 0.8	0.051
HOMA-IR	3.3 ± 3.6	4.2 ± 4.1	2.5 ± 2.8	<0.001
Progesterone (nmol/L)	2.8 ± 7.9	2.7 ± 10.0	2.8 ± 5.5	0.826
Testosterone (nmol/L)	1.7 ± 0.6	1.7 ± 0.7	1.7 ± 0.6	0.585
LH (mIU/mL)	10.6 ± 6.4	9.6 ± 5.5	11.4 ± 6.9	<0.001
FSH (mIU/mL)	6.1 ± 1.7	5.9 ± 1.6	6.3 ± 1.8	<0.001
LH/FSH ratio	1.8 ± 1.1	1.7 ± 0.9	1.9 ± 1.3	0.003
E2 (pmol/L)	269.6 ± 317.5	256.6 ± 325.0	278.8 ± 310.8	0.286
SHBG (nmol/L)	42.6 ± 30.7	32.9 ± 24.7	49.9 ± 32.6	<0.001
**Lipid profile**				
TC (mmol/L)	4.7 ± 1.1	4.9 ± 1.4	4.6 ± 0.7	<0.001
LDL-C (mmol/L)	3.0 ± 0.9	3.2 ± 1.1	2.8 ± 0.6	<0.001
HDL-C (mmol/L)	1.3 ± 0.4	1.1 ± 0.4	1.4 ± 0.3	<0.001
TG (mmol/L)	1.6 ± 0.9	2.1 ± 1.1	1.1 ± 0.4	<0.001
ApoA1 (g/L)	1.5 ± 0.3	1.4 ± 0.3	1.6 ± 0.3	<0.001
ApoB (g/L)	0.9 ± 0.3	1.0 ± 0.3	0.8 ± 0.2	<0.001
LP (mg/L)	130.1 ± 102.6	128.2 ± 95.8	131.8 ± 107.6	0.589

* *P values for comparisons between with dyslipidemia and without dyslipidemia*.

*Data are presented as mean ± SD or number (frequency)*.

*BMI, body mass index; HC, hip circumference; WC, waist circumference; SBP, systolic blood pressure; DBP, diastolic blood pressure; HOMA-IR, homeostasis model assessment for insulin resistance; LH, luteinizing hormone; FSH, follicle-stimulating hormone; E2, estradiol; SHBG, sex hormone-binding globulin; TC, total cholesterol; LDL-C, low-density lipoprotein cholesterol; HDL-C, high-density lipoprotein cholesterol; TG, triglyceride; ApoA1, apolipoprotein A1; ApoB, apolipoprotein B; LP, lipoprotein*.

Univariate logistic regression analysis showed that age, BMI, WC, HC, SBP, DBP, duration of infertility, glucose, insulin, and HOMA-IR were significantly associated with higher chance of dyslipidemia, while LH, FSH, and SHBG were significantly associated with lower chance of dyslipidemia (Table 2). By backward selection, multivariate logistic regression analysis showed that age (OR = 1.08, 95% CI: 1.03–1.13, *P* < 0.001), WC (OR = 1.03, 95% CI: 1.02–1.05, *P* < 0.001), insulin (OR = 1.004, 95% CI: 1.002–1.007, *P* < 0.001), FSH (OR = 0.90, 95% CI: 0.83–0.99, *P* = 0.022) and SHBG (OR = 0.98, 95% CI: 0.98–0.99, *P* < 0.001) were independent predictors for dyslipidemia in Chinese women with PCOS (Table 3).

**Table 2:** Univariate logistic analysis of the predictors for dyslipidemia.

	**Odds ratio**	**95% confidence interval**	***P*** **value**
Age (year)	1.09	1.04–1.13	<0.001
BMI (kg/m^2^)	1.17	1.13–1.21	<0.001
HC (cm)	1.06	1.05–1.08	<0.001
WC (cm)	1.06	1.05–1.07	<0.001
SBP (mmHg)	1.03	1.01–1.04	<0.001
DBP (mmHg)	1.02	1.00–1.03	0.049
Duration of infertility (month)	1.01	1.00–1.02	0.004
Current smoking	2.67	0.80–8.93	0.112
Current drinking	1.20	0.77–1.85	0.423
Insulin (pmol/L)	1.008	1.006–1.011	<0.001
Glucose (mmol/L)	1.15	1.01–1.31	0.042
HOMA-IR	1.21	1.14–1.28	<0.001
Progesterone (nmol/L)	1.00	0.98–1.02	0.826
Testosterone (nmol/L)	1.06	0.87–1.29	0.584
LH (mIU/mL)	0.95	0.93–0.97	<0.001
FSH (mIU/mL)	0.87	0.80–0.94	<0.001
E2 (pmol/L)	1.00	1.00–1.00	0.292
SHBG (nmol/L)	0.98	0.97–0.98	<0.001

*BMI, body mass index; HC, hip circumference; WC, waist circumference; SBP, systolic blood pressure; DBP, diastolic blood pressure; HOMA-IR, homeostasis model assessment for insulin resistance; LH, luteinizing hormone; FSH, follicle-stimulating hormone; E2, estradiol; SHBG, sex hormone-binding globulin*.

**Table 3:** Multivariate logistic analysis of the predictors for dyslipidemia by backward selection.

	**Odds ratio**	**95% confidence interval**	***P*** **value**
Age (year)	1.08	1.03–1.13	<0.001
WC (cm)	1.03	1.02–1.05	<0.001
Insulin (pmol/L)	1.004	1.002–1.007	<0.001
FSH (mIU/mL)	0.90	0.83–0.99	0.022
SHBG (nmol/L)	0.98	0.98–0.99	<0.001

*WC, waist circumference; FSH, follicle-stimulating hormone; SHBG, sex hormone-binding globulin*.

**Figure 2:**
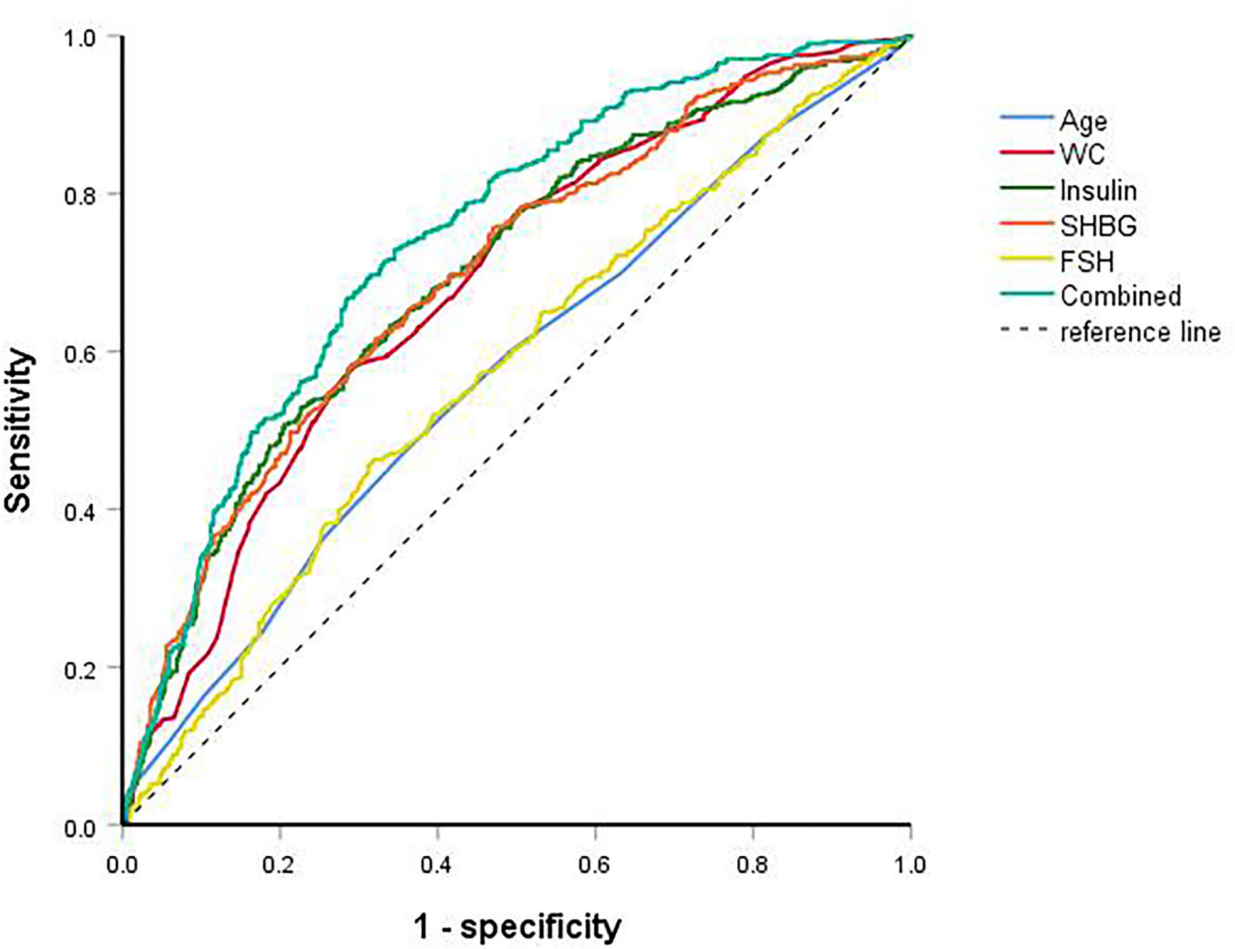
ROC curves of age, waist circumference, insulin, follicle-stimulating hormone, sex hormone-binding globulin and combined for dyslipidemia.

ROC curves were used to predict dyslipidemia (Figure 2). AUC of age, WC, insulin, FSH, and SHBG for dyslipidemia were 0.571, 0.683, 0.698, 0.574, and 0.698, respectively (Table 4). The cut-off points of age, WC, insulin, FSH, and SHBG were >28.5 years, >86.5 cm, >96.0 pmol/L, <5.6 mIU/mL, and <31.0 nmol/L, respectively. Sensitivity and specificity were listed in Table 4. When combined all of the above predictors, the AUC was up to 0.744 (95% CI: 0.713–0.775) for dyslipidemia.

**Table 4:** AUC, sensitivity, specificity for cut-off points of each predictor for dyslipidemia.

	**Cut-off value**	**Sensitivity**	**Specificity**	**AUC**	**95% confidence interval**
Age (year)	>28.5	47.2%	64.0%	0.571	0.534–0.608
WC (cm)	>86.5	58.1%	70.5%	0.683	0.650–0.717
Insulin (pmol/L)	>96.0	50.7%	79.6%	0.698	0.663–0.731
FSH (mIU/mL)	<5.6	69.1%	45.3%	0.574	0.537–0.611
SHBG (nmol/L)	<31.0	67.8%	61.7%	0.698	0.663–0.730

*AUC, The area under ROC curve; WC, waist circumference; FSH, follicle-stimulating hormone; SHBG, sex hormone-binding globulin*.

## Discussion

This study assessed the prevalence, pattern and predictors for dyslipidemia in Chinese women with PCOS. We found that prevalence of dyslipidemia is high in Chinese women with PCOS, and the most common type is low HDL-C. Age, WC, insulin, FSH, and SHBG were significant independent predictors for dyslipidemia. The overall predictability is good.

Several studies had reported the pattern and predictors for dyslipidemia in women with PCOS. One study reported that the prevalence of dyslipidemia was 76.1% and low HDL-C (57.6%) is the predominant lipid abnormality in Brazilian women with PCOS, and BMI had a significant impact on this abnormality (11). Another study found that the 72.4% of non-Hispanic women with PCOS had dyslipidemia and elevation in LDL-C was most frequent, and the significant predictors were age, insulin, and testosterone (19). In our study, the overall prevalence of dyslipidemia was 41.3% and low HDL-C was most common (26.9%). The low prevalence of dyslipidemia in Chinese women with PCOS might be explained be by the relatively lower BMI (15, 20).

Dyslipidemia is closely related to the occurrence and progress of CVD due to its role on atherosclerosis (21). TC, LDL-C and TG are atherogenic and represent risk factors of CVD, and LDL-C remains the primary target in lipid lowering therapy (22). HDL-C exerts athero-protective effects and prevent the CVD due to its role its role in reverse cholesterol transport (23) and favorable function on inflammation, oxidation, angiogenesis, and glucose homeostasis (24). Anti-inflammatory and antioxidant role of HDL-C is increasingly considered to be beneficial for reproductive health (25). Previous studies reported that HDL-C in follicular fluid has considerable antioxidative properties and is beneficial for the development of human oocytes and embryos (13, 26). Unfavorable serum lipid levels were also associated with worse reproductive outcomes in women undergoing assisted reproduction (12). Since low HDL-C was the most frequent type of dyslipidemia, strategies to improve HDL-C level might bring additional benefits for women with PCOS.

Some features have been reported to be associated with dyslipidemia. Aging is related to disorders of lipid metabolism through multiple pathways (27). WC is one of the most practical measurements for abdominal obesity, which contribute to the dyslipidemia and other metabolic traits of PCOS (28). Insulin resistance was associated with serum lipid level in women with PCOS (29). Our results confirmed that age, WC, and insulin were predictors for dyslipidemia in Chinese women with PCOS.

Some studies have evaluated the relation between FSH and lipid levels in postmenopausal women. One study found FSH was positively and linearly associated with TC and LDL-C (30). FSH may interact with its receptors in hepatocytes and reduce LDL receptor levels, which subsequently attenuates the endocytosis of LDL-C, resulting in an elevated circulating LDL-C level in postmenopausal women (31). On the contrary, we found that higher FSH level was associated with lower chance of dyslipidemia in women with PCOS. Under the regulation of FSH and its receptor, the oocyte relies on serum lipids from the maternal circulation to provide cholesteryl esters for granulosa cell steroidogenesis. We hypothesize that higher FSH level may stimulate the estrogen synthesis pathway, which lead to less lipid accumulation in circulation due to the increased synthesis of estrogen.

Our study also found protective role of SHBG on dyslipidemia in women with PCOS. Previous studies found that increased lipoprotein lipase activity was associated with better lipid metabolism, and a positive correlation between SHBG and lipoprotein lipase activity was observed (32). Since both lipid metabolism and SHBG production occur in the liver, the relationship between lipid and SHBG may reflect the changes of liver metabolism. SHBG may affect lipid metabolism by regulating insulin resistance. SHBG could affect glucose transporters and PI3K/AKT pathway (33, 34). Recent studies have found that lower SHBG is an independent risk factor for type two diabetes, and there is strong genetic evidence that SHBG is associated with the etiology of type two diabetes (35, 36). A previous study assessed SHBG levels in women with PCOS, and found that low SHBG levels were associated with gestational diabetes mellitus (37). Genetic studies also found that the causal relationship between SHBG, insulin resistance and diabetes is weak, indicating that the correlation was partially confounded rather than directly endowed by circulating SHBG (38).

Since the prevalence of dyslipidemia is rather high in women with PCOS, evaluation for lipid level is recommended. The current study showed that age, WC, insulin, FSH, and SHBG were predictors for dyslipidemia in women with PCOS. Age >28.5 years old, WC >86.5 cm, insulin >96.0 IU/L, FSH >5.6 mIU/mL, and SHBG <31.0 nmol/L were suggestive for lipid evaluation in Chinese women with PCOS.

Hyperandrogenism is regarded as a key element of the pathophysiology of PCOS, which is responsible for many clinical features of PCOS and reflect the severity of the syndrome. However, we didn't find that testosterone was a significant predictor for dyslipidemia. One study also found no correlation between testosterone and dyslipidemia in women with PCOS (39). We hypothesized that dyslipidemia may contribute more than traditionally thought in the development of PCOS.

Follicular microenvironment is the location for folliculogenesis, oocyte meiosis and steroidogenesis. Serum lipid metabolites could be transferred from circulation to follicular fluid, and directly contact with oocyte. Lipidomics analysis identified 32 differential lipids in the follicular fluid of PCOS women compared to control women (40). Previous study showed that high levels of serum lipid metabolic parameters were associated with increased number of oocytes retrieved in women with PCOS undergoing unstimulated natural cycles (13). We hypothesized that dyslipidemia might be an underlying cause of PCOS. The association between lipid metabolites in serum and follicular fluid, and the association between them and folliculogenesis, oocyte quality in women with PCOS still need more research in the future.

There are several strengths of this study. First, the women were recruited from different sites across different geographical regions in China mainland, which increased the generalizability of the cohort. Second, common clinical data were collected in a standardized method, blood samples were stored in a standard method and measured in a core laboratory. However, data of some participants were missing, which might decrease the statistical power. In addition, some characteristics of the subjects were lacking, such as physical activity, which might affect lipid metabolism. Additionally, the women didn't have a long-term follow-up and therefore we cannot evaluate the real cardiovascular health.

In conclusion, dyslipidemia was common in Chinese women with PCOS, and low HDL-C level was the predominant lipid abnormality. Older age, higher WC, higher insulin, lower FSH, and lower SHBG were predictive for dyslipidemia among Chinese women with PCOS.

## Data Availability Statement

The original contributions presented in the study are included in the article/supplementary material, further inquiries can be directed to the corresponding author/s.

## Ethics Statement

The studies involving human participants were reviewed and approved by First Affiliated Hospital, Heilongjiang University of Chinese Medicine. The patients/participants provided their written informed consent to participate in this study.

## Author Contributions

X-KW designed the study and critically revised the manuscript. W-YC and XL performed data analysis, drafted manuscript, interpreted the results, and revised the manuscript. All authors contributed to the article and approved the submitted version.

## Funding

This was funded by National Public Welfare Projects for Chinese Medicine (201107005 and 200807002), the National Key Discipline of Chinese Medicine in Gynecology during the year of 2009–2016 (JC200804), the Intervention for Polycystic Ovary Syndrome Based on Traditional Chinese Medicine Theory—Tian Gui Disorder (2011TD006), and the National Clinical Trial Base in Chinese Medicine Special Projects (JDZX2012036 and 2015B009) during the year of 2009–2016 for the First Affiliated Hospital, Heilongjiang University of Chinese Medicine, as well as the Heilongjiang Province Longjiang Schola Program to X-KW.

## Conflict of Interest

The authors declare that the research was conducted in the absence of any commercial or financial relationships that could be construed as a potential conflict of interest.

## Publisher's Note

All claims expressed in this article are solely those of the authors and do not necessarily represent those of their affiliated organizations, or those of the publisher, the editors and the reviewers. Any product that may be evaluated in this article, or claim that may be made by its manufacturer, is not guaranteed or endorsed by the publisher.
